# High-Level Expression of *Bacillus naganoensis* Pullulanase from Recombinant *Escherichia coli* with Auto-Induction: Effect of *lac* Operator

**DOI:** 10.1371/journal.pone.0078416

**Published:** 2013-10-23

**Authors:** Yao Nie, Wei Yan, Yan Xu, Wen Bo Chen, Xiao Qing Mu, Xinye Wang, Rong Xiao

**Affiliations:** 1 School of Biotechnology and Key Laboratory of Industrial Biotechnology, Ministry of Education, Jiangnan University, Wuxi, Jiangsu Province, China; 2 State Key Laboratory of Food Science and Technology, Jiangnan University, Wuxi, Jiangsu Province, China; 3 Center for Advanced Biotechnology and Medicine, Department of Molecular Biology and Biochemistry, Rutgers University, Piscataway, New Jersey, United States of America; New England BioLabs, United States of America

## Abstract

Pullulanase plays an important role in specific hydrolysis of branch points in amylopectin and is generally employed as an important enzyme in starch-processing industry. So far, however, the production level of pullulanase is still somewhat low from wide-type strains and even heterologous expression systems. Here the gene encoding *Bacillus naganoensis* pullulanase was amplified and cloned. For expression of the protein, two recombinant systems, *Escherichia coli* BL21(DE3)/pET-20b(+)-*pul* and *E. coli* BL21(DE3)/pET-22b(+)-*pul*, were constructed, both bearing T7 promoter and signal peptide sequence, but different in the existance of *lac* operator and *lacI* gene encoding *lac* repressor. Recombinant pullulanase was initially expressed with the activity of up to 14 U/mL by *E. coli* BL21(DE3)/pET-20b(+)-*pul* with IPTG induction in LB medium, but its expression level reduced continually with the extension of cryopreservation time and basal expression was observed. However, *E. coli* BL21(DE3)/pET-22b(+)-*pul* , involving *lac* operator downstream of T7 promoter to regulate foreign gene transcription, exhibited pullulanase activity consistently without detected basal expression. By investigating the effect of *lac* operator, basal expression of foreign protein was found to cause expression instability and negative effect on production of target protein. Thus double-repression strategy was proposed that *lac* operators in both chromosome and plasmid were bound with *lac* repressor to repress T7 RNA polymerase synthesis and target protein expression before induction. Consequently, the total activity of pullulanase was remarkably increased to 580 U/mL with auto-induction by *lac* operator-involved *E. coli* BL21(DE3)/pET-22b(+)-*pul*. When adding 0.6% glycine in culture, the extracellular production of pullulanase was significantly improved with the extracellular activity of 502 U/mL, which is a relatively higher level achieved to date for extracellular production of pullulanase. The successful expression of pullulanase with *lac* operator regulation provides an efficient way for enhancement of expression stability and hence high-level production of target protein in recombinant *E. coli*.

## Introduction

Pullulanase (pullulan-6-glucanohydrolase, EC 3.2.1.41) is a kind of enzyme acting on branched substrates, generally used for hydrolysis of glycogen and amylopectin, by cleaving the α-1,6-glucosidic linkages in amylaceous polysaccharides [1-3], and hence belongs to the glycosyl hydrolases (GHase) family 13 that is also termed as the α-amylase family [4]. The most important industrial application of pullulanase is the production of glucose and maltose syrups from starch hydrolysis. Because pullulanase specifically hydrolyzes the branch points in the amylopectin, whereas glucoamylase or β-amylase has only to hydrolyze the linear α-1,4-glucosidic linkages, so using pullulanase in combination with glucoamylase or β-amylase during saccharification process would allow for more efficient and rapid conversion reactions [5,6]. As an industrially important enzyme, therefore, pullulanase is generally employed together with other amylolytic enzymes (α-amylase, β-amylase, glucoamylase) to efficiently break down recalcitrant biomass into fermentable sugars for generating biofuels and other chemical commodities [7-9].

So far, pullulanases has been discovered and identified from various microorganisms, such as *Anaerobranca gottschalkii* [10], *Clostridium thermosulfurogenes* [11], *Geobacillus thermoleovorans* [12], *Klebsiella variicola* [13], *Raoultella planticola* [14], *Rhodothermus marinus* [15], *Thermotoga neapolitana* [16], and species of the genus *Bacillus* [17-19], and even the uncultured environment [20,21]. However, the employment of those wide-type strains for the production of pullulanase for industrial application is still limited due to low enzymatic activity [14,16]. Natural protein sources rarely meet the requirements for quantity and ease of isolation.

The occurrence of recombinant DNA technology and its application have enabled many proteins (or enzymes) to be produced in quantities that may otherwise be difficult to be obtained from natural sources [22]. That option also provides an opportunity for the heterologous expression of pullulanase-encoding genes [23]. Among many systems available for protein expression, the thoroughly characterized *Escherichia coli* remains one of the most attractive hosts with a fast growth rate at a high density in inexpensive media and the ability to over-synthesize the protein of interest [24-26]. Although a number of genes coding pullulanases have been cloned and expressed, the pullulanase expression level remains somewhat low and mostly reported as specific activity but not extracellular activity in broth [10,12,27]. In addition, in spite of the extensive knowledge on the genetics and molecular biology of *E. coli*, foreign gene could not always be expressed efficiently as a routine matter in *E. coli* [12,25,28,29]. 

To achieve high-level production of target protein, expression systems are ordinarily equipped with very strong promoters with the rationale that the more mRNA is produced, the more protein product should be made. Of the *E. coli* expression systems, the T7 promoter-based expression system is generally very powerful for high-level expression of recombinant protein due to the high activity of T7 RNA polymerase, whose expression level typically reaches up to 50% of the total cellular protein, and thus is superior to other *E. coli* expression systems [26,30]. However, only basal level of T7 RNA polymerase activity can lead to substantial expression of foreign protein even without inducer [31], and such basal expression of foreign protein, especially toxic proteins, would bring about negative effects on expression stability and consequent protein production [31-33]. Therefore, *lac* operator would be taken into account to tightly repress expression in the pre-induction phase for reducing the transcription level of target mRNA and also the basal expression level of foreign protein by blocking both the promoter for T7 RNA polymerase synthesis in the chromosome of *E. coli* and T7 promoter for target protein expression in the vector [31,34,35].

In this paper, we describe the heterologous expression of the gene (*pul*) encoding the pullulanase from *Bacillus naganoensis* JNB-1 (PUL) in recombinant *E. coli*. Pullulanase from *B. naganoensis* has been reported as an aciduric and thermoduric enzyme with the optimal activity at pH 4.5 and 60 °C, which is suitable for industrial starch hydrolysis process [36]. Therefore, this study was initiated to construct recombinant *E. coli* expression systems, to investigate the effect of *lac* operator element, and to achieve stable and high-level production of pullulanase in *E. coli*.

## Results and Discussion

### Construction of PUL expression systems concerning lac operator

**Figure 1:**
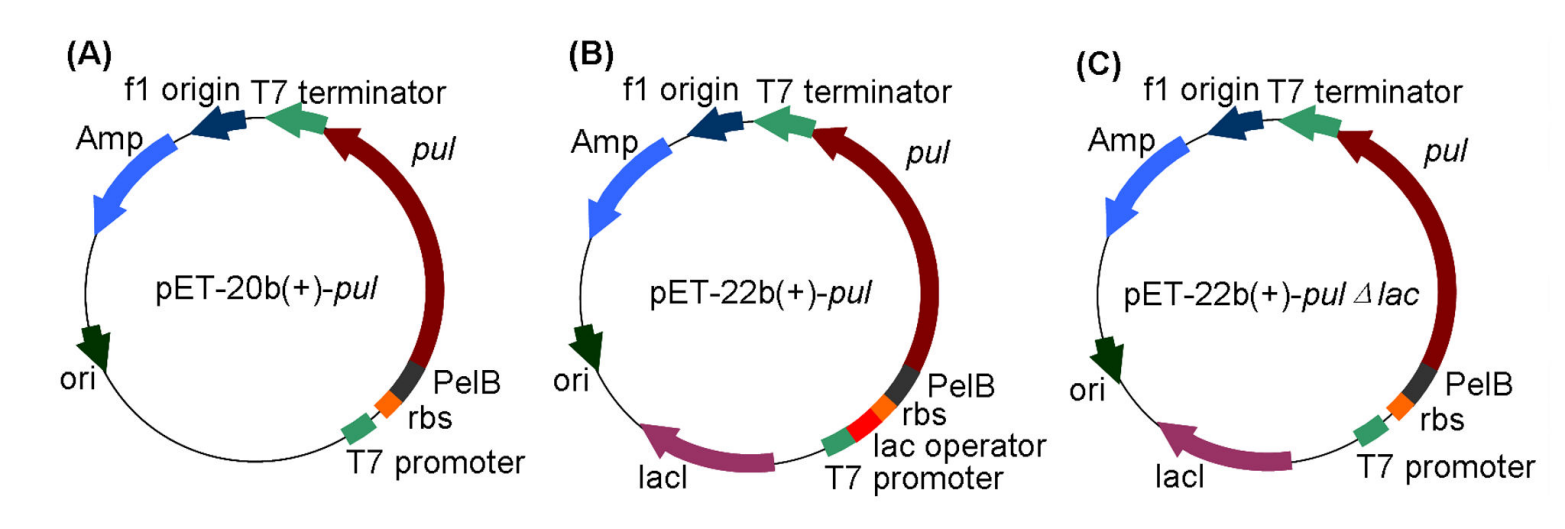
Constructed plasmid maps of (A) pET-20b(+)-pul, (B) pET-22b(+)-pul, and (C) pET-22b(+)-pulΔlac*.*

The pullulanase gene *pul* was amplified by PCR with the specific primers based on the pullulanase-encoding gene sequence (GenBank Accession No. JN872757), using genomic DNA of *B. naganoensis* as the template. The obtained open reading frame was 2,781 bp in length, encoding 926 amino acids with the predicted molecular weight of 101.4 kDa. To make the extracellular production of the target protein more feasible, pET-20b(+) and pET-22b(+) were selected as the candidate vectors both bearing T7 promoter and signal peptide sequence, but different in the existance of *lac* operator sequence and *lacI* gene encoding *lac* repressor. Recombinant plasmids harboring the pullulanase gene *pul*, pET-20b(+)-*pul* and pET-22b(+)-*pul*, were constructed by ligation to the vectors at *Bam*HI and *Xho*I restriction sites, respectively (Figure 1). 

To further confirm the effect of *lac* operator on the expression of target protein, the *lac* operator sequence in pET-22b(+)-*pul* was deleted to generate the mutant plasmid pET-22b(+)-*pul*Δ*lac*. Specific primers were designed to amplify the DNA fragment (5’-*Bgl*II site-T7 promoter-rbs-PelB signal peptide-*pul* gene-*Xho*I site-3’) in pET-20b(+)-*pul*, which contained the same functional sequences as those of pET-22b(+)-*pul*, except for the lack of *lac* operator sequence. After digested with *Bgl*II and *Xho*I, the resulted DNA fragment was inserted into pET-22b(+) and the mutant recombinant plasmid pET-22b(+)-*pul*Δ*lac* was obtained, whose sequence was the same as that of pET-22b(+)-*pul*, except that the former contained no *lac* operator sequence (Figure 1).

### Expression of PUL in LB medium

**Figure 2:**
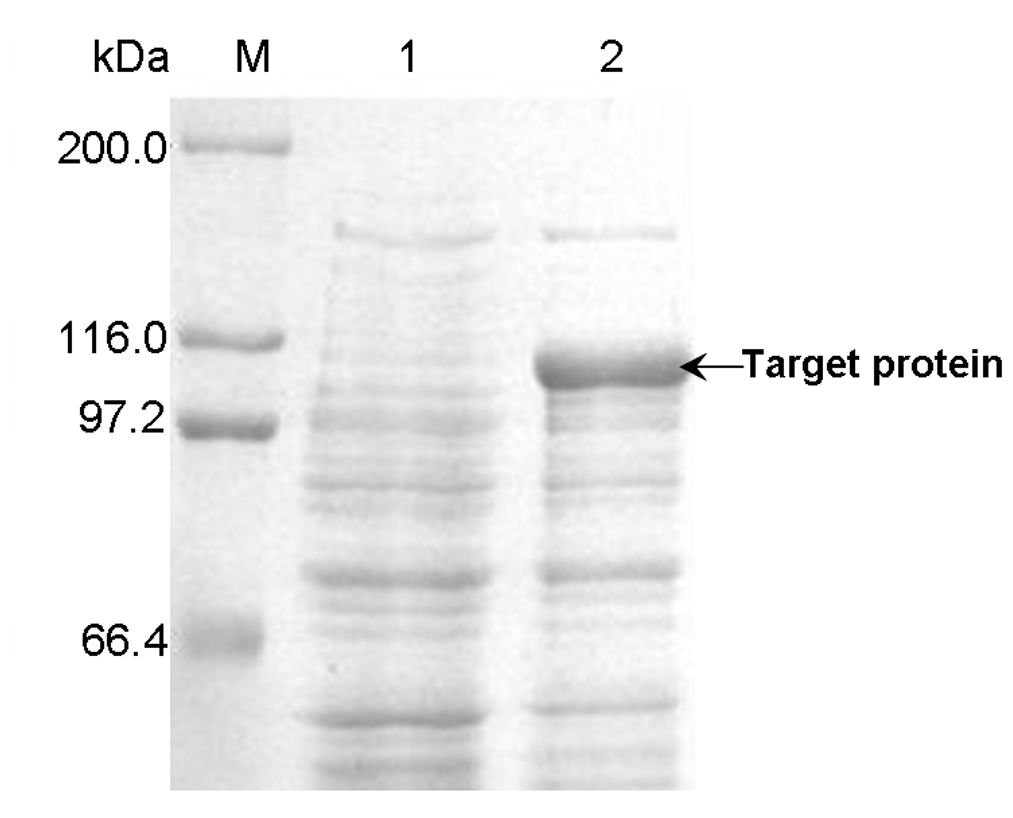
SDS-PAGE analysis of PUL expression in *E. coli* BL21(DE3)/pET-20b(+)-*pul* before and after degeneration. Lane M: protein molecular weight marker; Lane 1: total protein after degeneration; Lane 2: total protein before degeneration.

When pullulanase gene *pul* was expressed in LB medium, the phenomenon of unstable expression was firstly observed for the recombinant *E. coli* BL21(DE3)/pET-20b(+)-*pul*. By the IPTG-induction method in LB medium, the fresh transformant of *E. coli* BL21(DE3)/pET-20b(+)-*pul* initially expressed PUL with the pullulanase activity of up to 14 U/mL, possessing the optimal temperature of 62.5 °C and optimal pH value of 4.5, which would be an aciduric and thermoduric enzyme as the wild type and suitable for industrial starch hydrolysis process. However, when using the frozen glycerol stock of *E. coli* BL21(DE3)/pET-20b(+)-*pul*, less and even no detectable pullulanase activity occurred, namely degeneration of expression strain. From the SDS-PAGE of total protein fractions before and after degeneration (Figure 2), the optimal expression of PUL was obtained when inoculating the recombinant immediately after transformation, while cultivation from the frozen glycerol stock performed the decreased expression level of PUL obviously and even undetected level. In a further research, the pullulanase activity was detected from the recombinant without IPTG induction (data not shown), indicating the basal expression in *E. coli* BL21(DE3)/pET-20b(+)-*pul*. As reported, leaked expression of target protein frequently happens to *E. coli* expression systems, and basal expression in the pre-induction phase, especially for the T7 expression system, might be detrimental to the host and consequently lead to the issues involving instability of expression system and decreased target protein synthesis [31,32,37]. 

**Figure 3:**
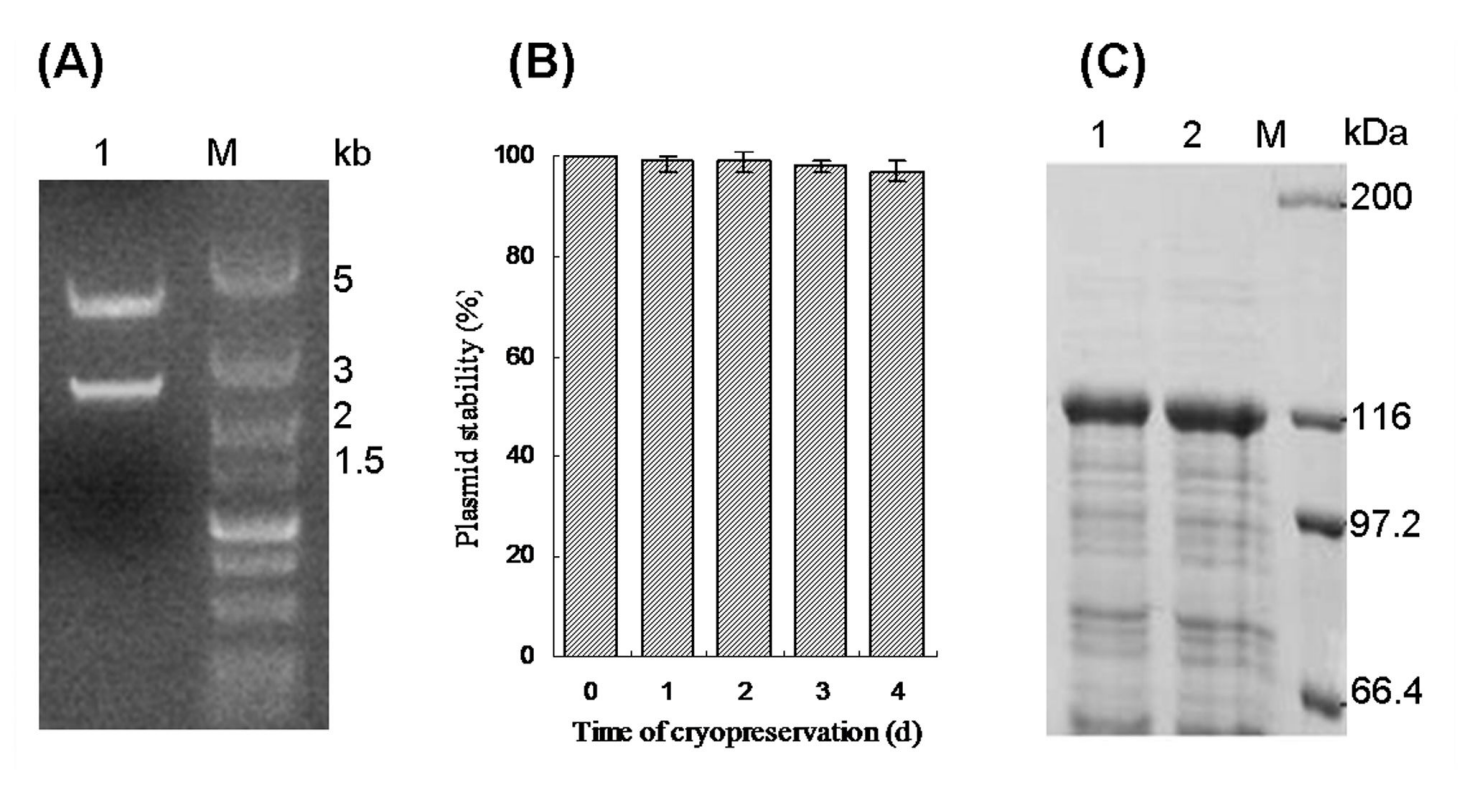
Analysis of loss and mutation of the recombinant plasmid from *E. coli* BL21(DE3)/pET-20b(+)-*pul*. (A) Restriction enzyme analysis of the recombinant plasmid pET-20b(+)-*pul*. Lane M: DNA Marker, Lane 1: double digestion of pET-20b-*pul*; (B) Plasmid stability during cryopreservation; (C) SDS-PAGE analysis of PUL expression in *E. coli* BL21(DE3)/pET-20b(+)-*pul* strain. Lane M: protein molecular weight marker, Lane 1: total protein of newly transformed strain, Lane 2: total protein of originally constructed strain.

To further address the contributing factors for decreased expression level of target protein from *E. coli* BL21(DE3)/pET-20b(+)-*pul* with time, a series of the possible issues were taken into account. At first, the recombinant plasmid was recovered from degenerated *E. coli* BL21(DE3)/pET-20b(+)-*pul* strain, followed by restriction enzyme analysis with *Bam*HI and *Xho*I. Then two DNA fragments were obtained, corresponding to the target gene and vector fragment from pET-20b(+) (Figure 3A). Although the obtained results were not quantitative, they indicated that plasmid loss alone could not account for the reduced level of expression. To address possible plasmid loss more directly, degenerated strains from same stock were plated on LB agar and LB agar containing ampicillin to investigate the ability of forming individual colonies with constant selective pressure for plasmid maintainence, respectively. It was found that the strains formed almost equivalent numbers of colonies on the plates whether or not ampicillin was present. In addition, the plasmid stability of the recombinant strain was also analyzed (Figure 3B). For most of the host cells, the loss of plasmid did not occur during the cryopreservation. Thus, plasmid loss would not be sufficient to be responsible for the observed decrease in protein expression. On the other hand, the recombinant plasmid from the degenerated strain was isolated and reintroduced into the competent *E. coli* BL21(DE3) cells. Then the level of protein production in newly transformed cells was similar to that of the original strain (Figure 3C). Therefore, plasmid loss or mutation would not be a significant cause of decreased target protein production with time. 

In our research, there was no clear growth disadvantage to *E. coli* BL21(DE3)/pET-20b(+)-*pul* strain, whether degenerated or not, as assessed by monitoring growth over time. Therefore, except for the lost and mutation of recombinant plasmid, mutation to the host cells, resulting in decreased level of functional T7 RNA polymerase, might be the predominant contributory factor for decreased production of target proteins [38]. As reported, even in the uninduced state and at low basal levels, expression of target protein would lead to polymerase mutations and loss of induction capability by resulted detrimental effect [38]. Hence, effective strategies to avoid loss of expression have been proposed previously based on preventing basal expression [38,31,33]. Therefore, the attempt to control the basal expression in the pre-induction phase should be critical for successful production of target protein [26,34,37].

**Figure 4:**
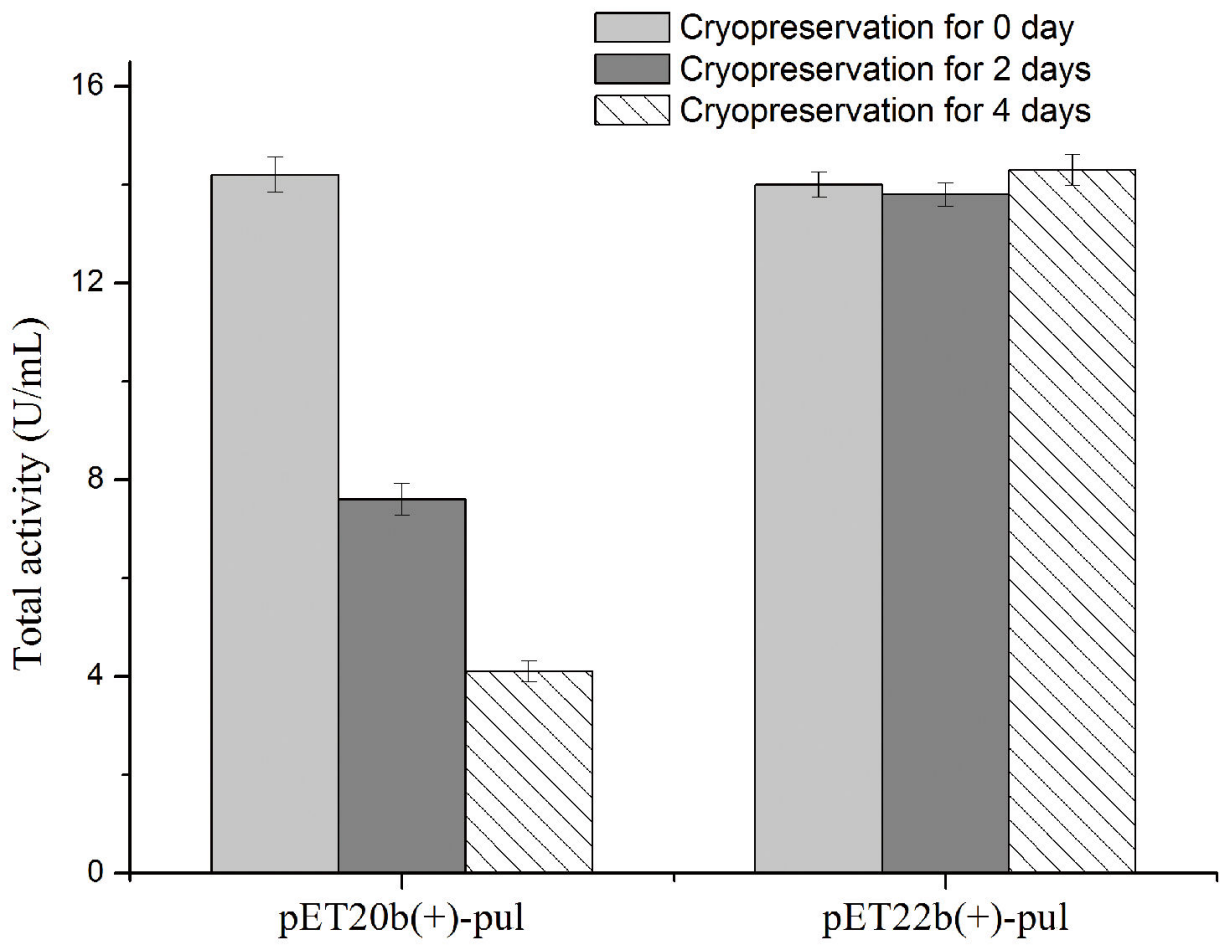
Effect of cryopreservation time of frozen glycerol stocks on expressed PUL activity in different recombinants. *E. coli* BL21(DE3)/pET-20b(+)-*pul* and *E. coli* BL21(DE3)/pET-22b(+)-*pul* were induced at 20 °C with 0.5 mM IPTG when cell turbidity (OD_600 nm_) reached 1.2. Fresh transformants were used for expression of the first time, and simultaneously seed culture of the fresh transformants was cryopreserved as frozen glycerol stocks used for the subsequent expressions.

Compared to *E. coli* BL21(DE3)/pET-20b(+)-*pul*, the recombinant *E. coli* BL21(DE3)/pET-22b(+)-*pul* involves a *lac* operator placed downstream of T7 promoter to regulate the transcription of foreign gene. When cultured in LB medium, *E. coli* BL21(DE3)/pET-22b(+)-*pul* exhibited no obvious pullulanase activity without IPTG induction, suggesting that there was no detected basal expression. Then the expression stability of *E. coli* BL21(DE3)/pET-22b(+)-*pul* was compared with that of *E. coli* BL21(DE3)/pET-20b(+)-*pul* for the cryopreservation stocks at different times. As shown in Figure 4, corresponding to the previous result, *E. coli* BL21(DE3)/pET-20b(+)-*pul* performed remarkable instability of PUL expression, where the PUL expression level reduced continually with the extension of cryopreservation time. By contrast, *E. coli* BL21(DE3)/pET-22b(+)-*pul* showed the expressed pullulanase activity consistently, indicating the stable expression of target protein. Considering the difference of the regulatory elements between these two expression systems, *E. coli* BL21(DE3)/pET-22b(+)-*pul* and *E. coli* BL21(DE3)/pET-20b(+)-*pul*, it was presumed that the expression stability of *E. coli* BL21(DE3)/pET-22b(+)-*pul* might attribute to the *lac* operator and the *lacI* gene in the pET-22b(+) vector.

### Effect of *lac* operator on PUL expression

As described above, the expression stability and final amount of the target protein in the involved expression systems would be mainly affected by the regulatory elements including *lac* operator placed downstream of T7 promoter and the *lacI* gene encoding *lac* repressor. The single copy chromosome of the host *E. coli* and the multicopy plasmid pET-22b(+) both contain the *lacI* gene providing constitutively sufficient *lac* repressor to saturate all of the *lac* operators in the cell, and *lac* repressor only bound to *lac* operator works to interfere with the transcription elongation [31]. Therefore, *lac* operator would play the role of improving the expression stability of the recombinant system. 

**Figure 5:**
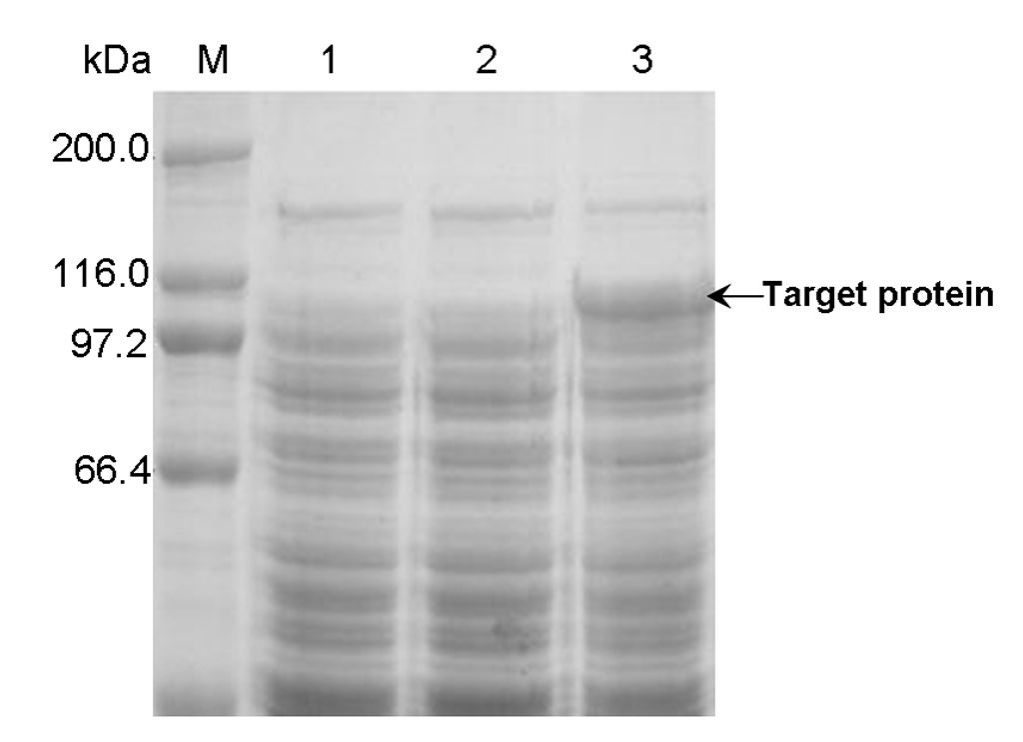
SDS-PAGE analysis of basal expression of PUL in different recombinants. Lane M: protein molecular weight marker; Lane 1: total protein of *E. coli* BL21(DE3)/pET22b(+); Lane 2: total protein of *E. coli* BL21(DE3)/pET22b(+)-*pul*; Lane 3: total protein of *E. coli* BL21(DE3)/pET22b(+)-*pul*Δ*lac*.

**Figure 6:**
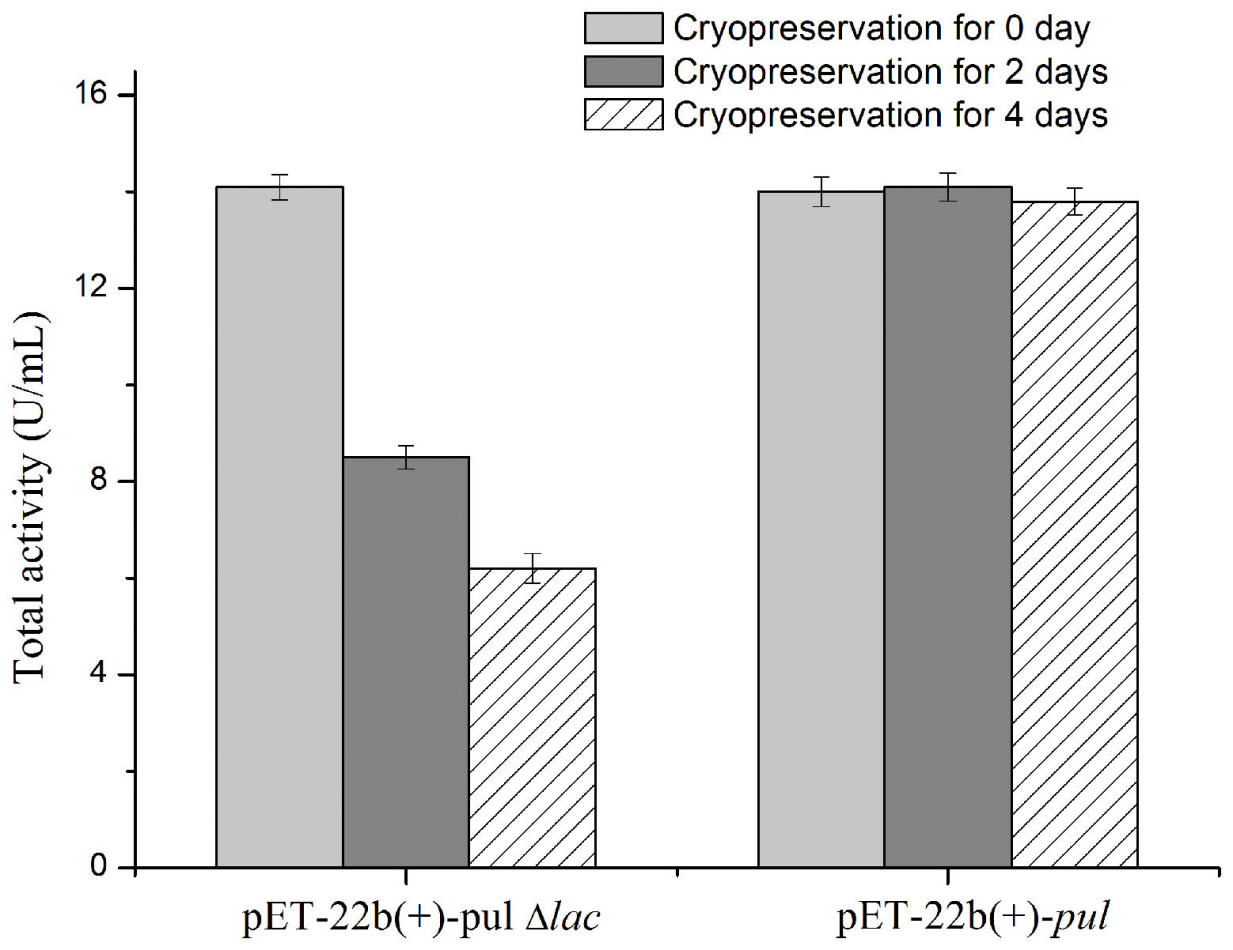
Effect of cryopreservation time of frozen glycerol stocks on expressed PUL activity in different recombinants. *E. coli* BL21(DE3)/pET-22b(+)-*pul* and *E. coli* BL21(DE3)/pET-22b(+)-*pul*Δ*lac* were induced at 20 °C with 0.5 mM IPTG when cell turbidity (OD_600 nm_) reached 1.2. Fresh transformants were used for expression of the first time, and simultaneously seed culture of the fresh transformants was cryopreserved as frozen glycerol stocks used for the subsequent expressions.

To circumstantiate the effect of *lac* operator, the mutant plasmid pET-22b(+)-*pul*Δ*lac* was constructed without the *lac* operator sequence, compared to pET-22b(+)-*pul*. The basal expression levels of the two systems, *E. coli* BL21(DE3)/pET-22b(+)-*pul* and *E. coli* BL21(DE3)/pET-22b(+)-*pul*Δ*lac*, were compared with LB medium under the same cultural conditions without induction. Protein band of PUL was found in the total protein sample of *E. coli* BL21(DE3)/pET-22b(+)-*pul*Δ*lac* (Figure 5), indicating that the deletion of *lac* operator in expression plasmid would generate detectable basal expression of foreign protein, which confirmed the contribution of *lac* operator to the tight transcription control of T7 expression system. The expression stability of frozen glycerol stocks of these two strains was also investigated (Figure 6). *E. coli* BL21(DE3)/pET-22b(+)-*pul*Δ*lac* lost its ability to synthesize PUL continually, while *E. coli* BL21(DE3)/pET-22b(+)-*pul* was able to maintain the expression stability, indicating *lac* operator actually improved the expression stability of *E. coli* BL21(DE3)/pET-22b(+)-*pul*. 

**Figure 7:**
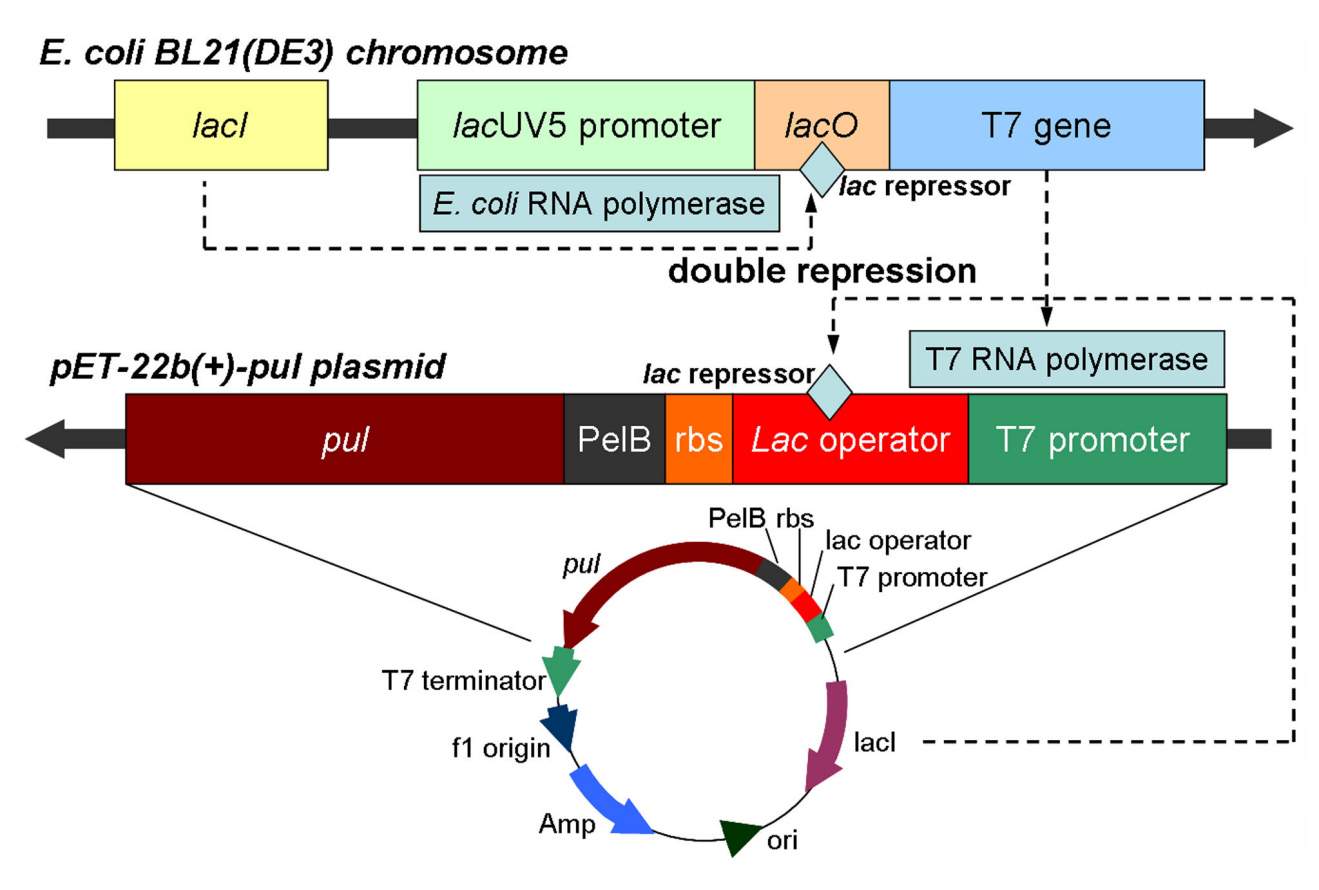
Scheme of double repression involving *lac* operator regulation for target protein expression.

In the host *E. coli* BL21(DE3), T7 gene encoding T7 RNA polymerase is under control of the inducible *lac*UV5 promoter and the *lac* operator in the chromosome DNA, while in the vector the gene encoding the desired protein is transcribed by the T7 promoter regulation, which is recognized by T7 RNA polymerase specifically [37]. Theoretically, in the absent of inducer, the binding of *lac* repressor to *lac* operator downstream of *lac*UV5 promoter greatly decreases the frequency of transcription elongation events by *E. coli* RNA polymerase, and hence the T7 gene stays silent in the uninduction phase [32,37]. Actually, however, T7 RNA polymerase would exhibit the basal activity and lead to the expression of target protein, namely the basal expression of target protein [31,37]. For *E. coli* BL21(DE3)/pET-22b(+)-*pul*Δ*lac*, because *lac* operator was deleted from the *pul* gene-inserted plasmid and no obstacle blocked T7 RNA polymerase to move towards the *pul* gene in the absent of inducer, even leaked expression of T7 RNA polymerase at a low level could initiated the transcription of the *pul* gene successfully. The resulted basal expression of PUL might be toxic and detrimental to the host, and consequently make the recombinant strain degenerated during cryopreservation and even cause growth inhibition in the pre-induction phase, which would lead to negative effects on the expression and accumulation of target protein in the induction phase after exponential growth of recombinant strain. In comparison, for *E. coli* BL21(DE3)/pET-22b(+)-*pul*, *lac* operator sequence following T7 promoter in “T7*lac*” promoter-involved plasmids also provides a binding site for *lac* repressor. Therefore, synthesis of T7 RNA polymerase initiated from chromosome of *E. coli* and expression of target protein generated from recombinant plasmid would be both repressed in the pre-induction phase. It was deduced that *lac* operator regulation would significantly affect the basal expression of foreign protein in the host strain, which might be detrimental to the host and consequently lead to the problems of system instability and negative effects on the accumulation of target protein. Then the double-repression strategy can be proposed to potentially reduce basal expression before induction and the detriment of foreign protein to the host, and enhance the production of target protein in induced cells (Figure 7) [31,37]. 

### Expression of PUL with auto-induction

**Figure 8:**
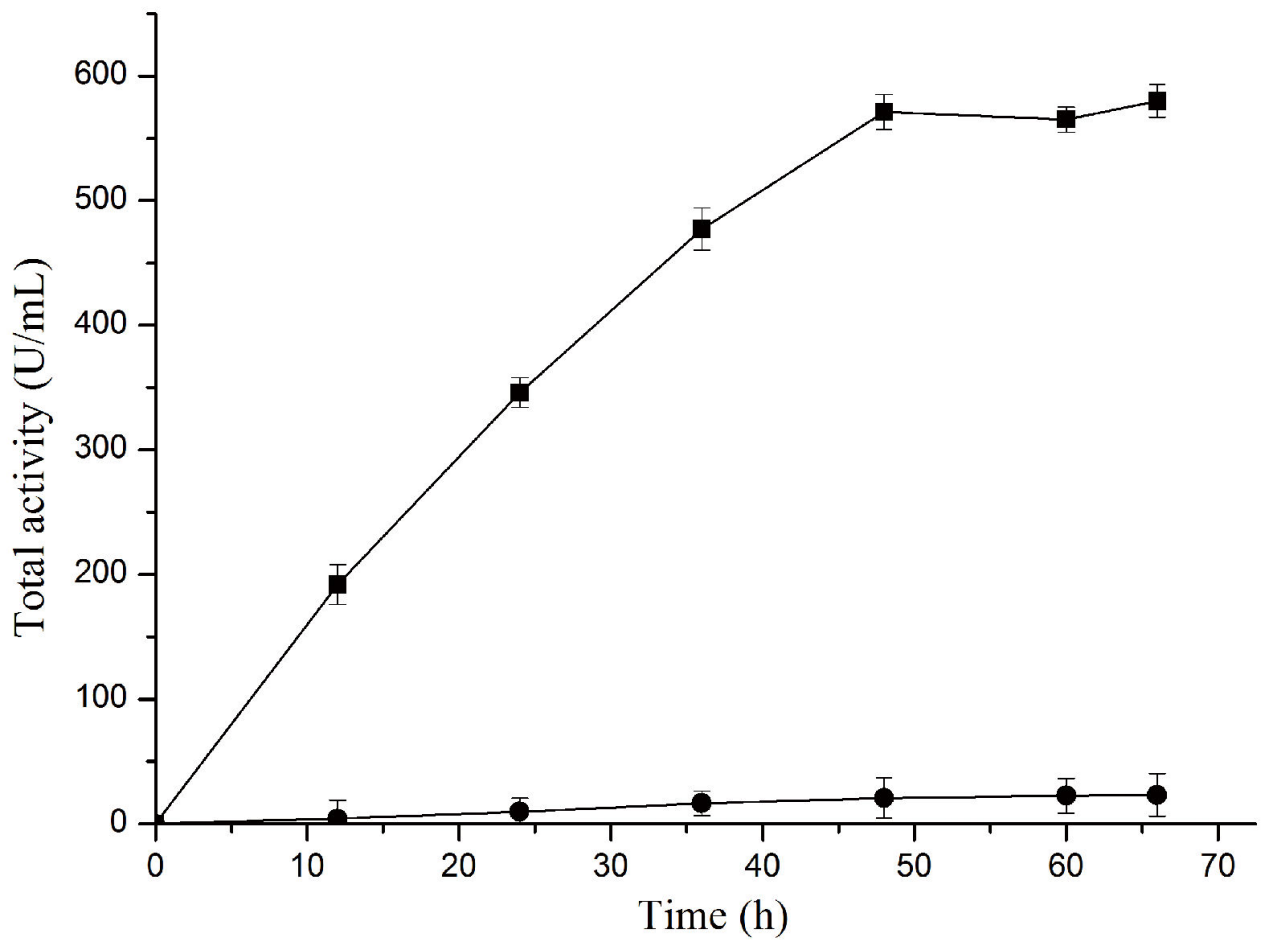
Expressed PUL activity profiles of different recombinants with auto-induction. The PUL activities were compared between the two recombinants, *E. coli* BL21(DE3)/pET-20b(+)-*pul* (circle) and *E. coli* BL21(DE3)/pET-22b(+)-*pul* (square).

**Figure 9:**
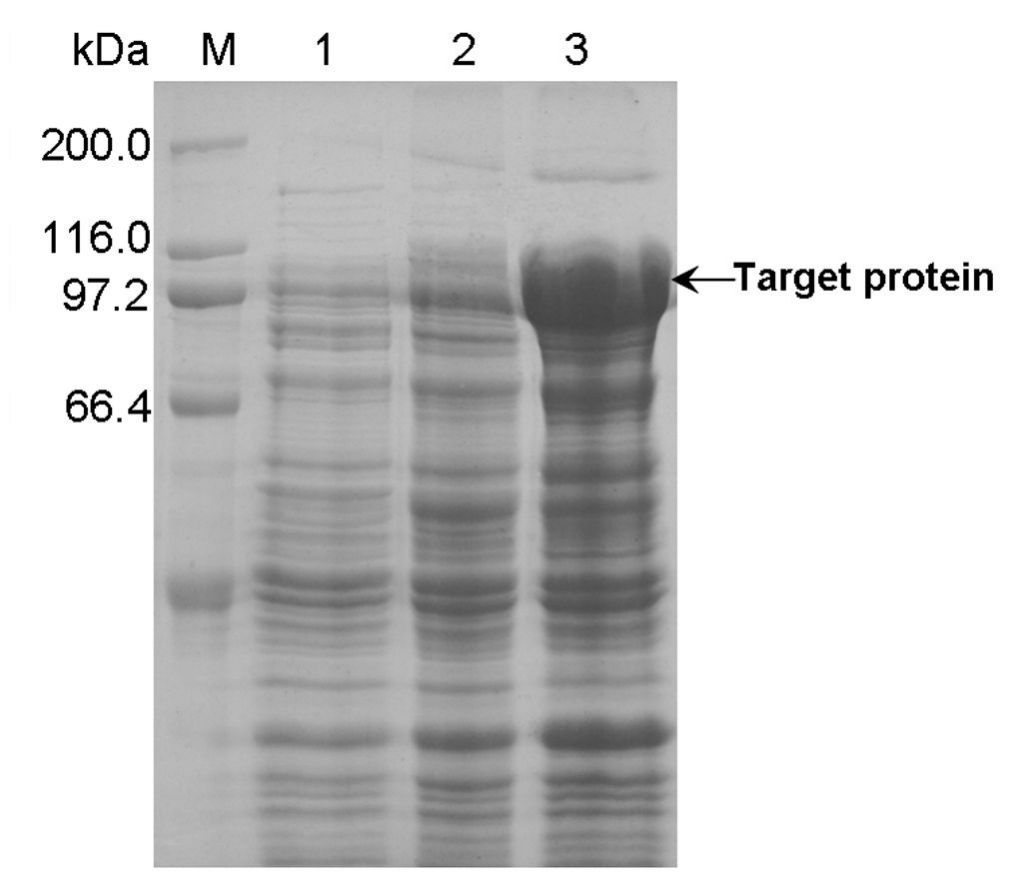
SDS-PAGE analysis of PUL expression in different recombinants with auto-induction. Lane M: protein molecular weight marker; Lane 1: total protein of *E. coli* BL21(DE3); Lane 2: total protein of *E. coli* BL21(DE3)/pET20b(+)-*pul*; Lane 3: total protein of *E. coli* BL21(DE3)/pET22b(+)-*pul*.

The auto-induction bacterial expression method has been proposed in these years and proved to be generally suitable for producing a wide range of proteins to a high yield [32]. Here the auto-induction method was adopted for production of PUL with the engineered strains *E. coli* BL21(DE3)/pET-22b(+)-*pul* and *E. coli* BL21(DE3)/pET-20b(+)-*pul* as the expression donators, respectively. As shown in Figure 8, the total activity of expressed PUL from *E. coli* BL21(DE3)/pET-22b(+)-*pul* increased obviously during the cultivation process with the final total activity of 580 U/mL, more than 40 folds of that in LB medium. By contrast, *E. coli* BL21(DE3)/pET-20b(+)-*pul* exhibited somewhat poor ability of PUL synthesis, with the final total activity of 23 U/mL merely. The SDS-PAGE analysis of the total proteins after expression (Figure 9) also showed that high-level expression of PUL was achieved in *E. coli* BL21(DE3)/pET-22b(+)-*pul* with auto-induction, much higher than that of *E. coli* BL21(DE3)/pET-20b(+)-*pul*.

Auto-induction method is developed from the regulation of bacteria in utilization of carbon and energy sources in the medium, based on the *lac* operon regulatory function. During the initial growth period, glucose is preferentially consumed as the carbon source and catabolite repression caused by the presence of glucose inhibits the uptake of lactose, while the depletion of glucose relieves the catabolite repression and leads to a shift in cellular metabolism to the import and consumption of lactose and glycerol, where lactose is converted to allolactose, the natural inducer of the *lac* operator, initiating the expression of target protein [32,39]. When using *E. coli* BL21(DE3)/pET-22b(+)-*pul*, in the early growth phase, glucose in the medium would block the induction by lactose, and double repression derived from the binding interactions between *lac* repressor and *lac* operator in both chromosome and plasmid would almost eliminate the basal expression of foreign protein and its detriment to the host, so that the recombinant strain could maintain its stability in cell growth and subsequent protein synthesis. When glucose was depleted, the utilization of lactose and glycerol would enable the cells to grow continually and induce the production of PUL. Therefore, with the combined strategy involving double-repression and auto-induction, the activity of recombinant pullulanase was significantly enhanced.

### Extracellular production of PUL by adding glycine

As known, heterologous protein expressed in secretory recombinant *E. coli* system is generally transported to the periplasmic space by the available signal peptide [40]. In this study, although the expression of PUL has been improved significantly, most of the target protein still accumulated in the periplasm fraction. Glycine, a common medium supplement, has been reported to induce modification of peptidoglycan structure in the cell wall and hence increase cell membrane permeability remarkably for enhanced secretion of desired protein from recombinant *E. coli* [41,42]. 

**Figure 10:**
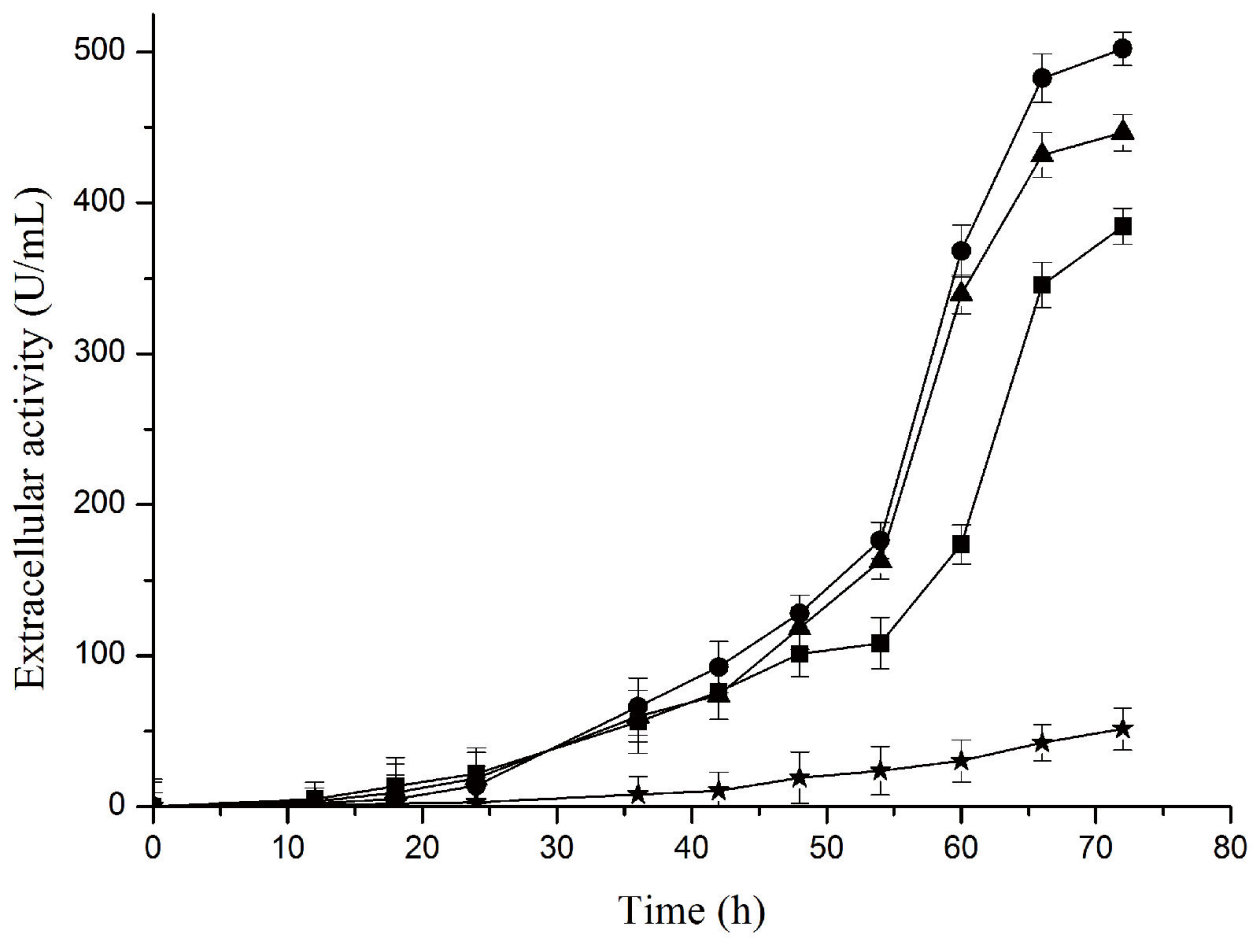
Effect of glycine concentration on extracellular activity of expressed PUL from *E. coli* BL21(DE3)/pET22b(+)-*pul*. Glycine was added in the auto-induction culture to the final concentration of 0 (star), 0.6% (circle), 0.9% (triangle), and 1.2% (square).

**Figure 11:**
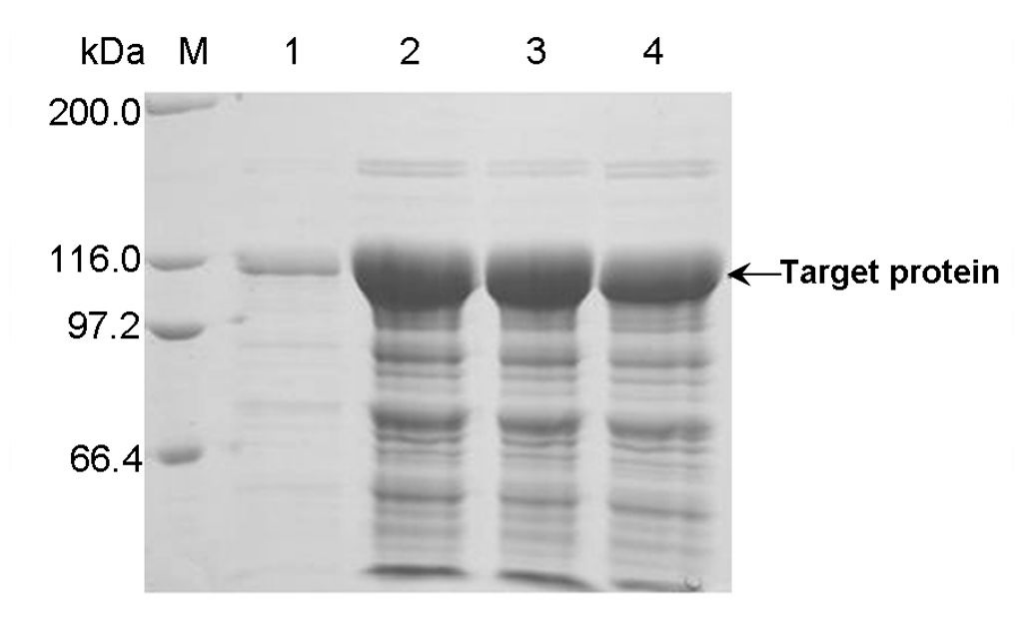
SDS-PAGE analysis of extracellular PUL production from *E. coli* BL21(DE3)/pET22b(+)-*pul* with glycine at different concentration. Lane M: protein molecular weight marker; Lane 1: extracellular fraction secreted without glycine; Lane 2: extracellular fraction secreted with 0.6% glycine; Lane 3: extracellular fraction secreted with 0.9% glycine; Lane 4: extracellular fraction secreted with 1.2% glycine.

To obtain the extracellular PUL, glycine was adopted as a kind of additive and supplemented into the culture to improve the extracellular production of PUL, and the effect of glycine concentration on protein secretion was also investigated. From both the respects of enzyme activity (Figure 10) and protein yield (Figure 11), extracellular production level of PUL from the *E. coli* BL21(DE3)/pET-22b(+)-*pul* was significantly improved when glycine was supplemented in the auto-induction culture, compared with that without glycine addition. Of the investigated glycine concentrations, the extracellular activity of PUL reached 502 U/mL with 0.6% glycine supplemented in the culture, almost 10 times of the control in the absence of glycine. To our knowledge, this is a relatively higher level achieved to date for heterologous expression and extracellular production of pullulanase.

## Conclusions

Pullulanase specifically hydrolyzing the branch points in the amylopectin is industrially important to be employed to efficiently break down biomass into fermentable sugars for generating biofuels and other chemical commodities. In this study, recombinant systems harboring the *B. naganoensis* pullulanase gene *pul* were constructed with T7 promoter and signal peptide sequence to facilitate the extracellular production of PUL in high yield. By comparing the expression of PUL in the involved recombinant systems, *E. coli* BL21(DE3)/pET-20b(+)-*pul* and *E. coli* BL21(DE3)/pET-22b(+)-*pul*, it was found that *lac* operator regulation would significantly affect the basal expression of foreign protein in the host strain, which might be detrimental to the host and consequently lead to the problems of system instability and negative effects on the accumulation of target protein. Then double-repression strategy was proposed to potentially reduce the basal expression before induction and the detriment of foreign protein to the host. Thus, *E. coli* BL21(DE3)/pET-22b(+)-*pul* was proved to be stable for PUL synthesis and high-level expression of PUL was achieved with auto-induction. In addition, glycine supplementation in culture further enhanced secretion and extracellular production of PUL with the extracellular activity of 502 U/mL. Therefore, this study would provide an efficient approach for enhancement of the expression stability of recombinant *E. coli* system and hence high-level production of the target protein. 

## Materials and Methods

### Strains, plasmids and materials

*B. naganoensis* JNB-1 stored in our lab was used as the source of the pullulanase-coding gene. *E. coli* strains JM109 conserved in our lab and BL21(DE3) purchased from the Novagen Company (USA) were used as the host for gene cloning and expression of target protein, respectively. The plasmids of pET-20b(+) and pET22b(+) were purchased from the Novagen Company (USA), both of which are controlled by T7 promoter and contain PelB signal peptide. The polysaccharide of pullulan for determination of pullulanase activity was purchased from Tokyo Kasei Kogyo Co., Ltd (Japan). Restriction endonucleases, DNA polymerase, and ligase were obtained from TaKaRa Biotechnology Co., Ltd (Dalian, China). The DNA primers and Plasmid Mini Kit were obtained from Sangon (Shanghai, China). All other chemicals are of analytical grade.

### Construction of recombinant plasmids

The open reading frame of the pullulanase-encoding gene was amplified using genomic DNA from *B. naganoensis* JNB-1 as the template. The specific pair of primers, PUL-F1 (5’-GAACAGGATCCAGATGGGAACACCACAAAC-3’) and PUL-R1 (5’-ATTCCCTCGAGTTTACCATCAGATGGGCT-3’), were synthesized based on the nucleotide sequence (GenBank Accession No. JN872757). The restriction sites *Bam*HI and *Xho*I were incorporated into the forward primer PUL-F1 and the reverse primer PUL-R1, respectively. The condition for PCR was as follows: one cycle at 95 °C for 5 min, 30 cycles at 95 °C for 1 min, 60 °C for 30 s, and 72 °C for 2 min 30 s, with an extra extension step at 72 °C for 10 min. Consequently an approximate 2.8 kb fragment was amplified. The PCR product was digested with *Bam*HI and *Xho*I, and then inserted into the vectors pET-20b(+) and pET-22b(+), resulting in the recombinant plasmids pET-20b(+)-*pul* and pET-22b(+)-*pul*, respectively. 

For the construction of pET-22b(+)-*pul*Δ*lac*, the DNA fragment was amplified using a pair of primers PUL-F2 (5’-GAACAAGATCTCGATCCCGC-3’) and PUL-R2 (5’-ATTCCCTCGAGTTTACCATCAGATGGGCT-3’), containing *Bgl*II and *Xho*I restriction sites, respectively. Using the plasmid pET-20b(+)-*pul* as the template, the PCR product of approximate 2.9 kb fragment was obtained, comprising the following elements *Bgl*II site-T7 promoter-rbs-PelB signal peptide-*pul* gene-*Xho*I site. After digested with *Bgl*II and *Xho*I, the resulted DNA fragment was inserted into vector pET-22b(+) to generate the desired recombinant plasmid pET-22b(+)-*pul*Δ*lac*. 

The obtained recombinant plasmids, pET-20b(+)-*pul*, pET-22b(+)-*pul*, and pET-22b(+)-*pul*Δ*lac*, were transformed into the expression host *E. coli* BL21(DE3) for pullulanase expression. The positive transformants were confirmed by PCR.

### Media and growth conditions

*B. naganoensis* JNB-1 was cultured at 30 °C for 2 days in the medium containing 0.25 g/L CaCl_2_·H_2_O, 0.5 g/L MgSO_4_·7H_2_O, 0.2 g/L (NH_4_)_2_SO_4_, 2 g/L yeast extract, 5 g/L glucose, and 3 g/L KH_2_PO_4_, with the pH adjusted to 5.0. Luria-Bertani (LB) medium (10 g/L tryptone, 5 g/L yeast extract, and 5 g/L NaCl) was used for *E. coli* cultivation and pullulanase expression for the study of system stability. Detection of basal expression was performed in LB medium without IPTG addition. A modified auto-induction medium [25,32], containing 10 g/L β-lactose, 0.5 g/L glucose, 50 g/L glycerol, 6.8 g/L KH_2_PO_4_, 0.25 g/L MgSO_4_, 10 g/L tryptone, 5 g/L yeast extract, 7.1 g/L Na_2_HPO_4_, 0.71 g/L Na_2_SO_4_, and 2.67 g/L NH_4_Cl, with the pH adjusted to 7.5-8.0, was used for high-level production of pullulanase. If necessary, ampicillin was added to media with the final concentration of 100 μg/mL. 

### Expression of PUL in LB medium

For protein expression in LB medium, *E. coli* BL21(DE3) harboring recombinant plasmid was inoculated into 5 mL LB medium in the presence of ampicillin (100 μg/mL) and incubated at 37 °C and 200 rpm overnight. Then the *E. coli* culture (0.5 mL) was transferred into a 250 mL Erlenmeyer flask containing 50 mL LB medium supplemented with ampicillin (100 μg/mL). The recombinant cells were cultured at 37 °C and 200 rpm, and when the culture turbidity (OD_600 nm_) reached 1.2, 0.5 mM IPTG was added to induce the heterologous expression. The culture was incubated for another 16 h at 20 °C and 200 rpm for the expression of target protein. For the investigation of basal expression, the cultivation and expression conditions were the same as the above, except for no IPTG added into the culture.

### Expression of PUL in auto-induction medium

For protein expression in auto-induction medium, *E. coli* BL21(DE3) harboring recombinant plasmid was inoculated into 5 mL LB medium in the presence of ampicillin (100 μg/mL) and incubated at 37 °C and 200 rpm overnight. Then the *E. coli* culture (2 mL) was transferred into a 250 mL flask containing 50 mL auto-induction medium supplemented with ampicillin (100 μg/mL). After cultivation at 37 °C and 200 rpm for the first 2 h, the culture was incubated at 20 °C and 200 rpm for another 70 h to produce the target protein. For comparing the expression level between the two recombinant systems *E. coli* BL21(DE3)/pET-20b(+)-*pul* and *E. coli* BL21(DE3)/pET-22b(+)-*pul*, fresh transformants were used for each cultivation batch to ensure the initial expression ability.

### Expression analysis and activity assay

The amount of target protein and the expression level were evaluated by combining the results involving activity assay and SDS-PAGE analysis of target protein. The samples for both activity assay and SDS-PAGE analysis were prepared with the same dilution so as to compare the amount of expressed target protein.

The culture was harvested by centrifugation at 12,000 rpm for 10 min at 4 °C, and the supernatant was colleted as the extracellular crude sample. The precipitate was washed twice with physiological saline and re-suspended in the same volume of physiological saline as that of the original culture. The suspension was subjected to ultrasonic for 15 min using a VCX750 cell sonifier. The insoluble debris was removed by centrifugation at 12,000 rpm for 30 min at 4 °C, and the supernatant was collected as the intracellular crude sample. The molecular mass and the amount of the recombinant enzyme were estimated by 9 % (w/v) sodium dodecyl sulfated-polyacrylamide gel electrophoresis (SDS-PAGE).

Pullulanase activity was assayed by measuring the aldehyde groups released during enzymatic reaction from a mixture consisting of pullulan solution and the diluted enzyme sample [13,14]. The reaction mixture, containing 0.1 mL 2 % (w/v) pullulan in 0.1 M sodium acetate buffer (pH 4.5) and 0.1 mL enzyme solution diluted with 0.1 M sodium acetate buffer (pH 4.5), was incubated at 60°C for 20 min. Then, the amount of released aldehyde groups was assayed with dinitrosalicylic acid (DNS) method by measuring the absorbance at 540 nm spectrophotometrically. One unit of pullulanase activity was defined as the amount of pullulanase that releases 1 μmol of aldehyde groups per min under the reaction conditions. Total activity was defined as the sum of extracellular and intracellular activity. Besides the standard method, the recombinant PUL was diluted with 0.1 M acetate buffer (pH3.0-6.0) to investigate the influence of pH values on the enzymatic activity. For the effect of temperature, enzyme samples were incubated at temperatures ranging from 40 to 70 °C, respectively, for 20 min. All the values of enzymatic activities shown in figures were averaged from three replicates with standard deviations, and significant differences (*p*<0.05) were measured.

### Analysis of plasmid stability

Analysis of plasmid stability was performed by calculating the ratio of colonies grown on selective and non selective LB plate medium [43].

### Effect of glycine on extracellular production of PUL

To enhance the secretion of pullulanase, glycine was added into the culture when the cultivation temperature was changed from 37 °C to 20 °C. The effect of glycine concentration on extracellular production of PUL was investigated by adding glycine into the culture with the final concentration of 0.6%, 0.9%, and 1.2%, respectively. The culture without glycine addition was set as the negative control.
